# Central Nervous System Tuberculosis Presenting With Multiple Ring-Enhancing Lesions: A Diagnostic Challenge

**DOI:** 10.7759/cureus.21819

**Published:** 2022-02-01

**Authors:** Nitin Desai, Rohini Krishnan, Lokesh Rukmangadachar

**Affiliations:** 1 Neurology, Saint Louis University School of Medicine, Saint Louis, USA

**Keywords:** intracranial tuberculoma, infectious disease, neurology, ring-enhancing lesions, meningitis, tuberculosis

## Abstract

*Mycobacterium tuberculosis *(TB) used to be one of the most widespread infections around the world. However, with improvements in sanitation, access to therapy, and increased public health efforts, TB has almost been eliminated from the developed world. While classically located in the lungs, infection can spread to distant sites from either late stage complications or as a sequelae from immunosuppression. In this paper, we present a case of a 53-year-old female who presented with nonspecific neurological complaints and multiple ring-enhancing lesions in the brain who was eventually diagnosed with central nervous system (CNS) tuberculosis after a lengthy workup despite following guideline-directed management. The purpose of this case report is to review an approach for ring-enhancing lesions and clinical considerations for central nervous system tuberculosis and add to the limited body of literature on the subject.

## Introduction

*Mycobacterium tuberculosis* (TB) infection, historically referred to as consumption, is a multifaceted disease with a variety of clinical presentations. Although the incidence of TB in the United States remains at an all-time low of 2.2 cases per 100,000 in 2020 [1], many patients are impacted significantly by a spectrum of primary, latent, and extrapulmonary manifestations notably including the skeletal system, lymphatic system, and central nervous system (CNS). Early diagnosis and treatment are imperative for better outcomes. As a result, clinicians must continue to consider TB within their differential diagnoses to ensure early diagnostic testing and rapid treatment to reduce the morbidity and mortality associated with clinical infection.

Meningeal involvement of TB is one of the least common presentations, accounting for only 4.4% of all extrapulmonary sites [2]. The degree of central nervous system involvement can vary depending on the length of the disease course, whether the patient is immunosuppressed or compromised, and the presence of comorbidities such as diabetes and chronic kidney disease.

The initial diagnosis of TB meningitis (TBM) is often challenging as the symptoms mirror other causes of meningitis, including fever, headache, nuchal rigidity, and vomiting. These findings can be further complicated by the formation of tuberculomas, which present as ring-enhancing lesions in the brain or spine. This makes it imperative to have a reliable schema for diagnosis as mortality from delayed treatment can be as high as 55%-75% [3]. Within this report, we will present a patient who received a guideline-driven diagnostic workup for her presenting symptoms; however, the case proved to be more challenging than initially anticipated. Although the prevalence of TB in the United States is low and the meningeal manifestations of TB infection is even lower, this case demonstrates the need for clinicians to consider TB as a potential cause early within the management of ring-enhancing lesions.

## Case presentation

A 53-year-old female with a past medical history of vulvar squamous cell carcinoma, hypertension, type 2 diabetes, and chronic tobacco use presented to the emergency department with a three-week duration of mild confusion and progressively worsening bi-occipital headache associated with photophobia, nausea, neck, and back pain.

The patient endorsed ongoing diplopia and fatigue but denied lightheadedness, vertigo, numbness, bowel/bladder incontinence, or symptomatic improvements with daily ibuprofen or acetaminophen. Her husband reported two incidences of slurred speech the week prior, and a visit to an outside hospital resulted in an unremarkable CT scan of the head with subsequent discharge with nausea medications.

**Figure 1:**
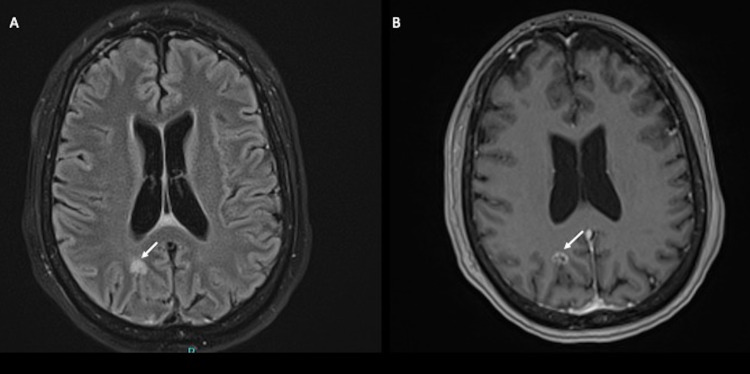
MRI of the brain with and without contrast. Panel A shows the axial T2 FLAIR sequence showing the T2 hyperintense lesion (arrow) in the right occipital lobe. Panel B shows the axial 3D T1 MPRAGE sequence showing the lesion taking up gadolinium contrast in a ring-enhancing pattern (arrow). MRI: magnetic resonance imaging; FLAIR: fluid-attenuated inversion recovery; MPRAGE: magnetization-prepared rapid acquisition gradient-echo

**Figure 2:**
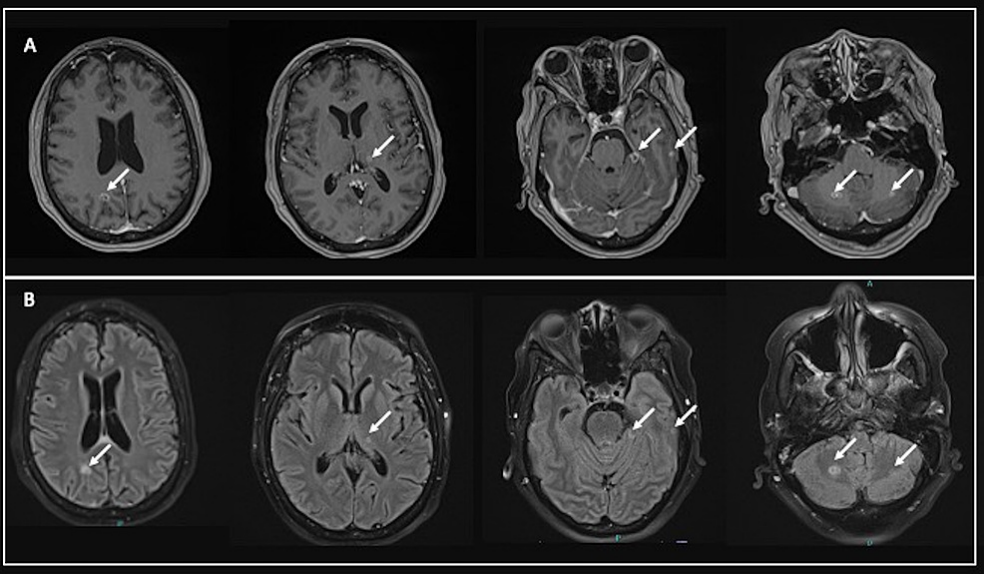
MRI of the brain with and without contrast. Panel A shows post-contrast axial T1 3D MPRAGE sequences demonstrating ring-enhancing lesions (arrows) in both supratentorial and infratentorial locations. Panel B shows axial T2 FLAIR sequences demonstrating hyperintense lesions (arrows) corresponding to the contrast-enhancing lesions in both supratentorial and infratentorial locations. MRI: magnetic resonance imaging; FLAIR: fluid-attenuated inversion recovery; MPRAGE: magnetization-prepared rapid acquisition gradient-echo

On presentation, the patient’s vital signs were within normal limits, and she was without focal neurological deficits. Emergent CT of the head did not show signs of acute intracranial processes; however, subsequent magnetic resonance imaging (MRI) with and without contrast revealed three supratentorial and five infratentorial ring-enhancing lesions (Figure 1 and Figure 2).

The patient was admitted, and additional diagnostic tests were performed. A CT of the chest, abdomen, and pelvis was obtained to screen for primary malignancy given suspicion for metastatic disease in the brain. CT of the pelvis demonstrated enlarged portocaval and retroperitoneal lymph nodes without evidence of neoplasm.

A lumbar puncture demonstrated lymphocytic pleocytosis (53%) with elevated protein in the cerebrospinal fluid (CSF) (Table 1). Additional laboratory tests for infections were performed; however, all resulted in a negative workup (Table 2). Rheumatologic workup was initiated; however, that too resulted in a negative workup (Table 3).

**Table 1:** Laboratory results of CSF workup. CSF: cerebrospinal fluid; Segs: segmented neutrophils; WBC: white blood cell; RBC: red blood cell; AFB: acid-fast bacillus; TB: tuberculosis

	Normal Range	Day 3 of Hospitalization (6/29)	Day 13 of Hospitalization (7/9)
Volume Fluid		2.0 mL	1.0 mL
Color Fluid	Colorless, Straw	Colorless	Colorless
Clarity Fluid	Clear	Clear	Clear
WBC Fluid	0–5/uL	72/uL	79/uL
RBC Fluid	0/uL	8/uL	60/uL
Xanthochromia Fluid	Negative	Negative	Negative
Protein CSF	15–45 mg/dL	142 mg/dL	103 mg/dL
Glucose CSF	40–70 mg/dL	99 mg/dL	50 mg/dL
Segs % Fluid		44%	14%
Lymphocytes % Fluid		53%	84%
Monocytes % Fluid		3%	2%
Additional Findings			
		Flow Cytometry CSF: No significant lymphoid population detected	Flow Cytometry CSF: No significant lymphoid population detected
		Fungus Stain: No yeast or hyphae seen	Fungus Stain: No yeast or hyphae seen
		AFB Smear: No AFB seen	AFB Smear: No AFB seen
		Culture CSF: Positive growth Mycobacterium tuberculosis (7/19)	Culture CSF + Gram Stain: Normal
		QuantiFERON TB Gold Plus: Positive	

**Table 2:** Infectious disease workup. Ab: antibody; IgG: immunoglobulin; CSF: cerebrospinal fluid; PCR: polymerase chain reaction; VDRL: venereal disease research laboratory test

Infectious Disease Panel	Values
Coccidioides Ab IgG Elisa CSF	0.2, Negative
Cryptococcus Antigen CSF	Negative
*Toxoplasma gondii* Antibody IgG	<3.0, Not Detected
*Enterovirus* by PCR	Not Detected
VDRL CSF	Nonreactive
Herpes Simplex Virus 1 PCR CSF	Nonreactive
Herpes Simplex Virus 2 PCR CSF	Nonreactive

**Table 3:** Rheumatological workup. IgG: immunoglobulin; ANA: antinuclear antibody; dsDNA: double-stranded DNA; ANCA: antineutrophil cytoplasmic antibodies; MPO: myeloperoxidase; SS-A: Sjögren’s syndrome antigen A; SS-B: Sjögren’s syndrome antigen B; ENA: extractable nuclear antigen; RNP: ribonucleoproteins

Variables	Normal Range	Results
IgG	767–1,590 mg/dL	1522 mg/dL
ANA IgG Antibody		None Detected
dsDNA Antibody	0–24 IU	2 IU
ANCA IgG Antibody	<1:20	1:160, High
MPO Antibody	0–19 AU/mL	1 AU/mL
Serine Proteinase 3	0–19 AU/mL	7 AU/mL
SS-A Antibody	0–40 AU/mL	3 AU/mL
SS-B Antibody	0–40 AU/mL	0 AU/mL
ENA Antibody	0–40 AU/mL	2 AU/mL
Complement C3	82–193 mg/dL	105 mg/dL
Complement C4	15–57 mg/dL	23 mg/dL
Centromere Antibody	0–40 AU/mL	1 AU/mL
Chromatin Antibody	0–19 Units	2 Units
Histone Antibody	0–0.9 Units	0.2 Units
Smith/RNP Antibody	19 Units or Less: Negative	2
SCL-70 Antibody	0–40 AU/mL	4 AU/mL

Over the following three days, the patient’s mental status worsened slightly with increased lethargy. We started her on empiric antibiotics for bacterial infection and rifampin, isoniazid, pyrazinamide, and ethambutol (RIPE) therapy with dexamethasone for suspected central nervous system tuberculosis after the TB QuantiFERON Gold test returned positive. CSF PCR for *M. tuberculosis* complex, however, returned negative, along with AFB smear and CSF cultures. Upon further questioning, the patient’s husband reported that the patient worked as an emergency department clerk at a regional hospital, and her most recent PPD test was about 10 mm a year ago alongside a negative chest X-ray.

Over the next week, the patients’ mental status improved with empiric therapy. Additional investigations were performed for definitive diagnosis. Evaluation for possible dissemination of vulvar cancer by biopsy showed a high-grade squamous intraepithelial lesion (VIN 3) with no evidence of invasive carcinoma. Biopsy of the portocaval lymph node did not show signs of malignancy. A second lumbar puncture obtained about a week after the first showed elevated protein (103 mg/dL) and normal glucose levels (Table 1). Subsequent testing showed negative gram/fungal stain, AFB smear, negative MTB PCR, and no significant lymphoid population on flow cytometry. CSF cultures still did not grow organisms at this point.

Empiric therapies were continued, and a stereotactic needle biopsy of the left temporal lesion was performed given the lack of definitive diagnosis. Histopathology showed necrotizing and non-necrotizing granulomatous inflammation with associated gliosis and was negative for gram stain, AFB smear, and culture of anaerobes or fungi. However, just in the nick of time, the initial CSF cultures from the first lumbar puncture grew *Mycobacterium* and confirmed the diagnosis of CNS tuberculosis four weeks after the patient’s presentation. The patient recovered well from the brain biopsy procedure without major complications and was discharged home with oral dexamethasone and RIPE therapy. She tolerated the treatment well and, at a three-month follow-up, was without any neurological issues. A repeat MRI of the brain showed decreased size of the previous ring-enhancing lesions and postoperative changes at the brain biopsy site.

## Discussion

Tuberculosis has been on a decline in developed Western nations; however, it is still rampant in other parts of the world, infecting close to 10 million people in 2020 [4]. In the United States, traditionally, the people who are at increased risk for TB are immigrants from high-risk areas, homeless persons, intravenous drug users, and healthcare workers. Primary infection occurs in the lungs and can spread hematogenously to distal sites including the nervous system. The onset of symptoms in immunocompetent patients can range between days and weeks and presents with typical findings of fever, headache, and vomiting. While TB meningitis (TBM) is only a fraction of extrapulmonary infections, it is by far the most lethal and demands proper workup with immediate treatment.

A challenging aspect of this case was the findings of multiple ring-enhancing lesions in an immunocompetent patient with no systemic symptoms. Etiologies for ring-enhancing lesions are generally in three categories: metastatic, infectious, or autoimmune. The most common cause of ring-enhancing lesions in the developed world is a metastatic neoplasm, while in the developing world, it is infectious. Magnetic resonance imaging is a powerful tool; however, the findings are oftentimes nonspecific, and differential diagnosis remains broad. In our case, the initial MRI noted multiple infratentorial and supratentorial ring-enhancing lesions, and based on the patient’s presentation, the differential diagnosis included metastasis, neurocysticercosis, toxoplasmosis, and granulomatous inflammation such as tuberculosis and sarcoidosis.

Neurocysticercosis is one of the most contending etiologies for multiple ring-enhancing lesions. It is a parasitic infection caused by *Taenia solium*, an intestinal pig tapeworm, through ingestion of undercooked food or contaminated water. The symptoms are divided based on intraparenchymal and extraparenchymal involvement, which include seizures, hydrocephalus, and manifestations of increased intracranial pressures such as headache, nausea, and altered mental status. The prevalence of neurocysticercosis in the United States is estimated to be around 0.5%-3% [5]. Radiological findings can be complex, and the presentation can vary based on the specific staging. A common radiographic finding for neurocysticercosis is ring calcifications of brain lesions. Given the low prevalence, lack of recent travel, and absence of classical findings on imaging, neurocysticercosis was low on our differential list.

*Toxoplasma gondii* is a common infectious cause for multiple ring-enhancing lesions in immunocompromised individuals with a CD4 count of less than 100 cells/mm^3^. Infection occurs with exposure to cat feces or undercooked meat and is concerning during pregnancy as it can affect the fetus. Classical image findings on CT show hypodense lesions that enhance with contrast and perilesional vasogenic edema that can cause mass effect [6]; lesions can not only be seen in the basal ganglia but also be found in the frontal and parietal lobes [7]. Our patient had lesions in both infratentorial and supratentorial regions without evidence of edema on CT, which is relatively nonspecific. Toxoplasmosis in immunocompetent individuals is a rare phenomenon [8], and with no history of pet exposure, cerebral edema on imaging, and an eventual negative toxoplasma Ab test, we were able to reasonably rule out this diagnosis in our clinical case.

CNS metastasis should be a high-priority differential if the patient has any history of cancer or other risk factors. Metastatic brain cancer has a higher occurrence than primary neoplasms, with the most common cancers including lung, breast, and melanomas. Typical CNS metastatic presentation depicts parenchymal involvement with multiple bilateral lesions across the gray-white junction. The treatment of metastatic lesions is dependent on tumor burden with the source of neoplasm and consists of a combination therapy of surgery, radiation, and chemotherapy. Our patient’s history of vulvar squamous cancer and smoking history prompted a workup to exclude neoplasm. Metastatic vulvar squamous cancer usually travels to the liver, lungs, and bone; however, there have been a handful of case reports showing cerebral metastasis [9-11]. The results of both biopsy of the vulva and negative whole-body PET scan made metastasis an unlikely cause of the lesions in our patient.

Tuberculosis was always a top contender from the inception of the case and only got more paramount as other differentials were ruled out. One facet that added to the perplexing nature of this case was the lack of consensus from the diagnostic testing. Our patient had a positive QuantiFERON Gold (sensitivity: 92%) [12] and negative chest X-ray and CT. Initial CSF analysis was not indicative of tuberculosis infection. CSF AFB stain (sensitivity: 45%-75%) and PCR (sensitivity: 98%) [13] were both unremarkable. Despite limited supportive diagnostic findings, we decided to start empiric therapy based on clinical suspicion with RIPE and corticosteroids since waiting for a confirmatory test would have worsened outcomes and increased mortality. Cultures are the gold standard; however, it can take up to two to four weeks on average to yield any result for fungal infections [14] and up to six weeks for tuberculosis infection. In situations with clinical suspicion for central nervous system tuberculosis, it would be reasonable to start therapy if all other diagnostic testing and imaging were negative for other etiologies.

The ultimate decision to conduct a brain biopsy was based on the lack of confirmation and confidence from other diagnostic tests. It yielded necrotizing and non-necrotizing granulomatous inflammation with associated gliosis and stained negative for AFB and fungus. Sarcoidosis, another close mimicker of tuberculosis, cannot be ruled out based on this description of histology. Confirming tuberculosis bacilli by PCR or culture from the biopsy specimen or another CNS source is required for the definitive diagnosis of CNS tuberculosis. In our case, cultures from the original lumbar puncture returned positive, finally securing the diagnosis of central nervous system tuberculosis. From here, we can postulate that the ring-enhancing lesions we saw are tuberculomas, which are space-occupying lesions seen in about 1% of cases of TBM [15]. Lesions classically occur in the parietal or frontal lobe in adults and infratentorial regions in children and show basal meningeal enhancement. Lesions can be symptomatic, leading to neurological deficits, or remain silent and usually disappear after initiation of RIPE therapy, with adjunct corticosteroids to reduce edema, just as we saw with our patient.

This case report is meant to highlight the importance of considering tuberculosis as an infectious cause of ring-enhancing lesions. Although meningeal manifestations of TB are rare, this case highlights the importance of screening for risk factors of TB while collecting a history of present illness and ordering laboratory works early within the course of a CNS infection with ring-enhancing lesions, especially when there is increased clinical suspicion. Other infectious causes of ring-enhancing lesions should be explored as well, as discussed above.

## Conclusions

This case report demonstrates the importance of a thorough workup to diagnose ring-enhancing lesions. The differentials remain broad for ring-enhancing brain lesions and should be guided by patient factors and demographics and refined as new information evolves from laboratory investigations. This case demonstrated that it is important to continue to consider TB infection as a potential cause of ring-enhancing lesions although the prevalence is low in the United States. When suspecting central nervous system tuberculosis, medical therapy should be initiated immediately while awaiting confirmation, as delayed treatment could lead to worse patient outcomes.

## References

[1] (2021). Centers for Disease Control and Prevention: Tuberculosis — United States, 2020. https://www.cdc.gov/mmwr/volumes/70/wr/mm7012a1.htm.

[2] (2021). Centers for Disease Control and Prevention: Reported Tuberculosis in the United States, 2019. https://www.cdc.gov/tb/statistics/reports/2019/national_data.htm.

[3] Bourgi K, Fiske C, Sterling TR (2017). Tuberculosis meningitis. Curr Infect Dis Rep.

[4] (2021). World Health Organization: Global Tuberculosis Report 2021. https://www.who.int/teams/global-tuberculosis-programme/tb-reports/global-tuberculosis-report-2021.

[5] Cantey PT, Coyle CM, Sorvillo FJ, Wilkins PP, Starr MC, Nash TE (2014). Neglected parasitic infections in the United States: cysticercosis. Am J Trop Med Hyg.

[6] Lee GT, Antelo F, Mlikotic AA (2009). Best cases from the AFIP: cerebral toxoplasmosis. Radiographics.

[7] Gupta RK, Lufkin RB (2001). MR imaging and spectroscopy of central nervous system infection.

[8] Basit KA, Nasir S, Vohra E, Shazlee MK (2018). Toxoplasmosis in an immunocompetent patient. Pak J Med Sci.

[9] Jaeger A, Biermann M, Prieske K (2020). Cerebral metastasis in recurrent squamous cell carcinoma of the vulva: case report and review of the literature. Arch Gynecol Obstet.

[10] Vázquez JP, Cobo SL, Antón FM, Asado AC, Vidart JA, Coronado P, Díaz-Rubio E (2007). Brain metastasis and carcinomatous meningitis from vulvar squamous cell carcinoma: case report. Eur J Gynaecol Oncol.

[11] Dursun P, Ayhan A, Tarhan NC, Coban G, Kuscu E (2009). Cerebellar metastasis in squamous cell vulvar carcinoma. Arch Gynecol Obstet.

[12] Detjen AK, Keil T, Roll S, Hauer B, Mauch H, Wahn U, Magdorf K (2007). Interferon-gamma release assays improve the diagnosis of tuberculosis and nontuberculous mycobacterial disease in children in a country with a low incidence of tuberculosis. Clin Infect Dis.

[13] Tang YW, Meng S, Li H, Stratton CW, Koyamatsu T, Zheng X (2004). PCR enhances acid-fast bacillus stain-based rapid detection of Mycobacterium tuberculosis. J Clin Microbiol.

[14] Ghodbane R, Raoult D, Drancourt M (2014). Dramatic reduction of culture time of Mycobacterium tuberculosis. Sci Rep.

[15] Mohammadian M, Butt S (2019). Symptomatic central nervous system tuberculoma, a case report in the United States and literature review. IDCases.

